# Association of IgA antiphospholipid antibodies with hypercoagulability and inflammation in antiphospholipid syndrome

**DOI:** 10.1007/s11239-026-03316-1

**Published:** 2026-05-18

**Authors:** Isabella Constantini Soares de Andrade, Luísa Reis Figueiredo Pinto, Silmara Aparecida de Lima Montalvão, Marina Pereira Colella, Gabriela Goés Yamaguti-Hayakawa, Joyce Maria Annichino-Bizzacchi, Erich Vinícius De Paula, Fernanda Andrade Orsi

**Affiliations:** 1 Hematology and Hemotherapy Center, University of Campinas (UNICAMP), Campinas, Brazil; 2 School of Medical Sciences, University of Campinas (UNICAMP), Rua Tessália Vieira de Camargo, 126 - Cidade Universitária, Campinas, 13083-887 SP Brazil

**Keywords:** Antiphospholipid syndrome, Antiphospholipid antibodies, IgA antibody, Inflammation, Thrombosis

## Abstract

**Abstract:**

Although IgA isotype testing does not enhance diagnostic accuracy of antiphospholipid syndrome (APS), recent studies suggest that IgA aCL/aβ2GPI are associated with systemic lupus erythematosus (SLE) and thrombosis, contributing to thromboinflammatory responses and potentially serving as prognostic markers. This study’s objective is to determine whether IgA aCL/aβ2GPI are associated with coagulation and inflammatory responses, as well as a high-risk thrombotic APS (t-APS) profile. A total of 81 patients with t-APS were included. IgA aCL and IgA aβ2GPI were measured using chemiluminescence. Coagulation and inflammatory markers (TNF-α, IL-6, IL-8, IFN-α, TF, VWF, ADAMTS13) were measured by ELISA, and then, their levels and activity compared between IgA-positive and negative patients. The association of IgA and relevant outcomes (antiphospholipid [aPL] profile, recurrent thrombosis, SLE), was assessed by logistic models adjusted for age and sex. IgA aCL or aβ2GPI positivity was observed in 24 patients (29.6%), mostly with both antibodies (*n* = 23). IgA positivity was not associated with demographics, comorbidities or pregnancy complications, but correlated with triple aPL positivity (48% vs. 13%, *P* = 0.004) and higher risk of progression to SLE (17% vs. 0%, *P* = 0.004). IgA-positive patients showed significantly elevated TNF-α, IL-8, and TF levels (*P* = 0.03, *P* = 0.03 and *P* = 0.05, respectively). In conclusion, IgA aCL/aβ2GPI positivity is associated with triple aPL positivity, progression to SLE, and altered thromboinflammatory markers, thus highlighting their association with a pronounced thromboinflammatory APS profile. This emphasizes the association of IgA antibodies with a high-risk serological profile and the potential utility of IgA testing in APS.

*Clinical trial registration:* This study is not a clinical trial that requires registration.

**Graphical abstract:**

**Figure:**
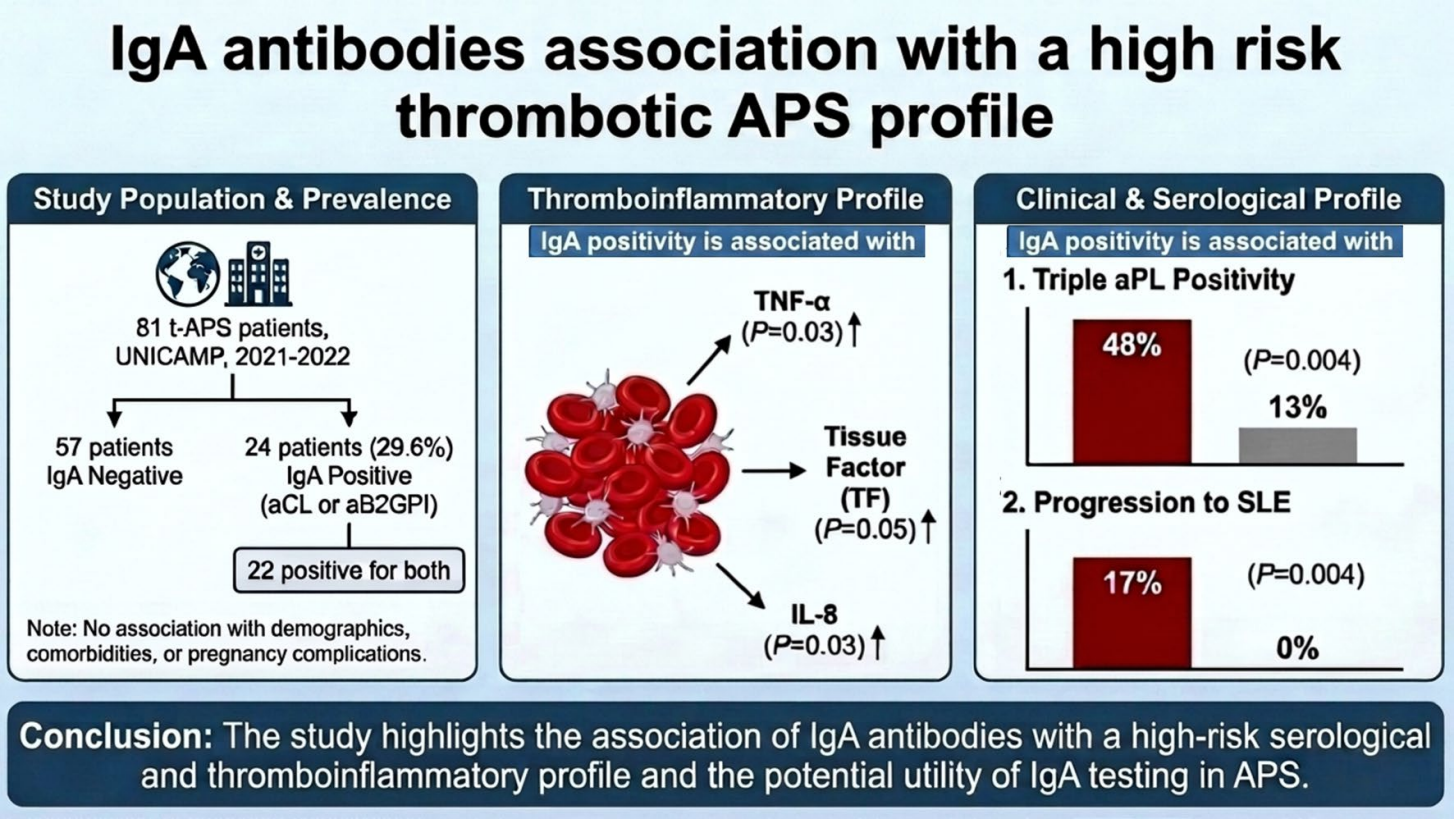

## Introduction

Laboratory diagnosis of antiphospholipid syndrome (APS) requires the detection of at least one of the three “criteria” antiphospholipid antibodies (aPL): lupus anticoagulant (LAC), IgG or IgM anticardiolipin (aCL), and IgG or IgM anti-β2 glycoprotein I (aβ2GPI) [1]. Although IgA aCL and IgA aβ2GPI have been associated with thrombotic manifestations of APS (t-APS), isolated IgA positivity is uncommon, and current evidence indicates that testing for IgA isotypes does not improve the diagnostic accuracy of APS [2].

Thrombotic complications related to APS are clinically heterogeneous, involving both arterial and venous sites [3]. They range from microvascular to large-vessel thrombosis and have a variable response to anticoagulation therapy [3]. The most widely accepted predictor of adverse outcomes in t-APS is triple positivity for aPL (aPL-TP), defined as the concurrent presence of LAC, aCL and aβ2GPI [4]. Besides, LAC positivity and a high score in Global Antiphospholipid Syndrome Score (GAPSS) are also considered [1, 5]. However, detection of aPL-TP can be challenging, particularly due to the need for temporary withdrawal of anticoagulation when testing for LAC, and additional prognostic markers are needed [6]. In this context, IgA antibodies may serve as prognostic markers in APS [7].

Recently, IgA aβ2GPI antibodies were associated with the diagnosis of systemic lupus erythematosus (SLE) [8], resulting in the inclusion of IgA aCL and IgA aβ2GPI antibodies as part of the classification criteria of SLE [9]. In APS, IgA aCL and IgA aβ2GPI are predominantly associated with thrombosis, rather than with other clinical manifestations of the disease [10]. A recent study demonstrated that IgA aβ2GPI are associated with thrombotic APS manifestations by binding to specific B2GPI epitopes, underscoring their pathogenic potential [11]. Additionally, IgA-immune complexes can trigger thromboinflammatory responses, such as the release of inflammatory cytokines and neutrophil extracellular traps [12].

In this context, the aim of this study is to investigate whether IgA positivity for aCL or aβ2GPI is associated with a high risk t-APS profile. We also sought to evaluate whether IgA aCL and IgA aβ2GPI are associated with increased coagulation and inflammatory responses in t-APS.

## Methods

### Patients selection

We included a cohort of t-APS patients treated at the Hematology and Hemotherapy Center at the University of Campinas, Brazil between 2021 and 2022. Inclusion criteria comprised diagnosis of APS and history of at least one thrombotic episode. Patients who did not fulfill the classification criteria for APS, who had APS with obstetric complications only or patients without previous thrombosis were excluded. One hundred fifteen patients diagnosed with t-APS were attended to at the outpatient unit during the enrollment period. Out of them, eighty one patients agreed to participate in the study.

### Patients diagnosis and management

APS diagnosis relied on the presence of persistently positive aPL combined with a history of thrombosis or obstetric complications, according to 2006 Sidney Criteria [13]. Persistent positivity for aPL was defined as persistent positive LAC; persistent positive IgG or IgM aCL at moderate to high titles (> 40 GPL or MPL) or persistent positive (> the 99th percentile) IgG/IgM anti-beta2 glycoprotein 1 (aβ2GPI), at two distinct times, with an interval of at least 12 weeks [13]. The laboratory tests were performed according to the international guidelines of the International Society of Thrombosis and Hemostasis (ISTH) [14]. aPL were tested 3 to 6 months after the thrombotic event, when it was considered safe to stop anticoagulation according to thrombosis treatment guidelines [15].

LAC were detected in plasma samples using two assays based on different principles; diluted Russell’s viper venom time (dRVVT) and silica clotting time (SCT). Screening and confirmatory tests were considered positive when the results were above the local cut-off value (99th percentile). Solid phase aPL (aCL IgG and IgM, and aβ2GPI IgG and IgM) were tested in patient serum by ELISA with cardiolipin or B2GP1 as antigen (Sigma-Aldrich, USA). A calibration curve and commercial controls were used; samples were tested in duplicate. The local cutoff value for aβ2GPI was determined by the 99th percentile, the cut-off value for aCL was > 40 GPL/ MPL [13, 14].

Thrombotic events were confirmed by imaging exams, such as ultrasound (US), computerized tomography (CT), magnetic resonance (MR) or ventilation/perfusion lung scan, according to the site of thrombosis. If clinical suspicion of recurrent venous thrombosis, new exams were performed and the results were compared with those of the last available examination. Recurrent venous thrombosis was diagnosed in cases in which a previously fully compressible segment (contralateral or ipsilateral) was no longer compressible or when there was an increase in the residual thrombosis. New arterial thrombosis was diagnosed when there were symptoms of ischemia and new abnormalities on imaging examinations (CT or MR). Diagnosis of myocardial infarction depended on the alterations of electrocardiogram and cardiac enzymes. All patients underwent prolonged anticoagulant treatment with warfarin; patients with arterial thrombosis received anticoagulation combined with antiplatelet agents.

### Quantification of IgA aCL and IgA aβ2GPI

Patients’ blood was collected at the enrollment for the study and stored at − 80 °C. IgA aCL or aβ2GPI were quantified in patients serum by the chemiluminescent immunoassay (QUANTA Flash^®^ Cardiolipin IgA and “QUANTA Flash^®^ anti β2GPI IgA; Inova Diagnostics), using BIO-FLASH Chemiluminescent Analyzer (Inova Diagnostics). We use the cut-off values established by the manufacturer (Inova Diagnostics).

### Quantification of coagulation factors and inflammatory markers

Serum levels of interferon -alpha (IFN-α), tumor necrosis factor-alpha (TNF-α), interleukins (IL)-6 and − 8 and TF and ADAMTS13 activity were measured using the following commercially available ELISA kits: VeriKine Human IFN-alpha ELISA kit (PBL Assay Science, Piscataway Township, NJ, USA), IL-6 Quantikine HS and TNF-αQuantikine HS (R&DSystems, Minneapolis, MN, USA), Human IL -8 ptEIATM (BD Biosciences Pharmingen, San Diego, CA, USA), IMUBIND^®^ Tissue Factor ELISA (BioMedica Diagnostics, USA) and Technozym^®^ ADAMTS-13 Activity (Technoclone, Austria), respectively. Plasma levels of von Willebrand factor (VWF) were measured using HemosIL von Willebrand Factor Activity assay. Components complement C3 and C4 were measured by a quantitative turbidimetric assay performed at the hospital central laboratory. The assays were performed according to the manufacturer’s instructions. Technicians were not aware whether the samples were from patients or controls.

### Statistical analysis

Categorical variables were expressed as absolute numbers and percentages. Numerical variables with normal distribution were expressed as mean and standard deviation. Numerical variables that were not normally distributed were expressed as median and interquartile (25th and 75th percentiles).

First, we determined the prevalence of IgA isotypes among t-APS patients included in this study. Next, we compared demographic parameters and clinical manifestations between IgA-positive and IgA-negative patients using Fisher’s exact test for categorical variables and Mann Whitney (2-group comparison) test for continuous variables. To assess the association between IgA isotype positivity and “criteria” aPL profile, we divided the patients into those with single positivity for these antibodies (LAC, IgM/IgG aCL or IgM/IgG aβ2GPI), double positivity (LAC plus IgM/IgG aCL or aβ2GPI; or IgM/IgG aCL plus aβ2GPI) and triple positivity (LAC plus aCL plus aβ2GPI). IgA aCL/aβ2GPI serum concentrations were compared between these subgroups using Kruskal-Wallis test.

Finally, we evaluated the correlation of IgA positivity and the following markers of inflammation and coagulation: TNF-α, IL-8, IL-6, INF-alpha and TF circulating levels, VWF and ADAMTS13 activity. Regression analysis, adjusted for age, sex and diagnosis of SLE, were performed to evaluate the association of IgA with aPL profile and inflammatory/coagulation markers. Statistical analysis was performed using SPSS (version 25, IBM, USA).

## Results

### IgA positivity and clinical characteristics of the patients

A total of 81 t-APS patients were included in the study. The patients’ demographic and clinical features are described in Table 1. The cohort was composed of 59 (72.8%) women and 22 (28%) men. The most prevalent comorbidity was dyslipidemia (*n* = 33, 40.7%), followed by hypertension (*n* = 28, 34.6%) and obesity (*n* = 15, 18.5%). The first thrombotic event was venous thrombosis in 56 patients (69%) and 37 (45.6%) patients had recurrent thrombosis. Pregnancy complications related to APS (abortion, fetal loss, preeclampsia or premature delivery) occurred in 55.8% of women with a history of pregnancy. In terms of “criteria” aPL positivity, 28 patients (34.5%) were single positive for LAC, 18 (22%) were double positive for LAC and either aCL or aβ2GPI and 17 patients (21%) were triple positive.

**Table 1:** Demographic and clinical characteristics at the date of inclusion in the study

Patients with t-APS *n* = 81	
*Gender*	
Women, n(%)	59 (72.8)
Male, n(%)	22 (27.2)
*Ethnicity*	
White, n(%)	69 (85.2)
Indigenous, n(%)	5 (6.2)
Black, n(%)	6 (7.4)
*Comorbidities*	
Obesity, n(%)	15 (18.5)
Hypertension, n(%)	28 (34.6)
Diabetes, n(%)	5 (6.2)
Dyslipidemia, n(%)	33 (40.7)
Chronic kidney disease, n(%)	8 (9.9)
*Pregnancy, n(%)*	43 (72.9)
Obstetric Complications*, n (%) of pregnancies ending in abortion, fetal loss or other complications	24 (55.8)

Abbreviations: t-APS, thrombotic antiphospholipid syndrome; Values are expressed in numbers and percentage. Age is expressed in years. *self-reported obstetrical complications: 43 women with t-APS had at least one pregnancy

Overall, 24 patients (29.6%) tested positive for IgA aCL (*n* = 23) or IgA aβ2GPI (*n* = 23), of whom 22 were positive for both antibodies. Eight patients (34.7%) with positive IgA aCL were negative for IgM and IgG aCL, while 4 patients (17.4%) with positive IgA aβ2GPI were negative for IgM and IgG aβ2GPI. Three IgA-positive patients (13%) had previously tested positive for LAC only. As shown in Table 2, IgA positivity was not associated with sex, age at diagnosis, ethnicity, comorbidities or pregnancy complications.

**Table 2:** Demographic and Clinical characteristics according to IgA aCL/aβ2GPI positivity

	IgA aCL/aβ2GPI positive *n* = 24		IgA aCL/aβ2GPI negative *n* = 57		*P*
Women, *n* (%)	17	(70.8)	42	(73.7)	0.79
Age at diagnosis, n (IQR)	37.7	(31-53.5)	45.1	(31-55.1)	0.79
Time elapsed since the last thrombotic event (months) median (IQR)	9.2	(7.2–17.5)	8.8	(6.3–15.0)	0.43
*Comorbidities*					
Obesity (BMI ≥ 30 kg/m2)	4	(16.5)	13	(22.9)	0.61
Hypertension, n (%)	8	(33.3)	20	(35.1)	0.74
Diabetes, n (%)	3	(12.5)	2	(3.5)	0.21
Dyslipidemia, n (%)	12	(50.0)	21	(36.8)	0.47
Chronic kidney disease, n(%)	4	(16.7)	4	(7.0)	0.12
Smoking	4	(16.7)	17	(29.8)	0.24

Abbreviations: aCL anticardiolipin (IgM or IgG). aβ2GPI anti beta 2 glycoprotein I. APS, antiphospholipid syndrome; Age is expressed in years; BMI body mass index. BMI data was missing in some patients and controls. IQR, interquartile range. P value < 0.5 was calculated using Mann-Whitney test. *self-reported obstetrical complications: 43 women with t-PAPS had at least one pregnancy

Table 3 demonstrates the distribution of IgA positivity by the type of thrombotic manifestation. Venous thrombosis was the index thrombotic event in 83.3% of the IgA-positive patients and 63.2% of the IgA-negative patients (*P* = 0.06). Recurrent thrombosis was similar between IgA-positive and -negative patients, since 54.2% of IgA positive patients and 40.4% of IgA negative patients had a history of multiple thrombosis (*P* = 0.32). The median time from t-APS diagnosis to thrombosis recurrence was also similar among groups, being 10.9 years (IQR: 7.9–13.8) among IgA positive and 8.5 years (IQR: 5.6–14.1) among IgA negative patients (*P* = 0.15).

Also, Table 3 demonstrates that, while IgA positivity was not associated with secondary APS at diagnosis, progression to SLE (among those initially diagnosed as primary APS) was more frequent among IgA positive patients than in IgA negative patients (17% vs. 0, *P* = 0.004).

**Table 3:** Clinical presentation of patients with IgA aCL/aβ2GPI positivity

	IgA aCL/aβ2GPI positive *n* = 24		IgA aCL/aβ2GPI negative = 57		*P*
*APS classification*					
Primary APS, n (%)	11	(45.8)	36	(63.2)	0.15
Secondary APS, n (%)	13	(54.2)	21	(36.8)	
*Site of the first thrombosis*					
Venous Thrombosis, n (%)	20	(83.3)	36	(63.2)	0.06
Arterial Thrombosis, n (%)	4	(16.7)	21	(36.8)	
*Thrombosis recurrence, n (%)*	13	(54.2)	23	(40.4)	0.32
*Site of recurrence*					
Venous Thrombosis, n (%)	12	(66.7)	15	(53.6)	0.38
Arterial Thrombosis, n (%)	6	(33.3)	13	(46.4)	
*Use of aspirin or anticoagulant at the time of recurrence, n (%)*	6	(40.0)	9	(42.9)	0.9
Obstetric Complications*, n (%) of pregnancies ending in abortion, fetal loss or other complications	5	(41.7)	19	(61.2)	0.16
*Triple positivity, n (%)*	10	(47.6)	7	(12.7)	0.004
ANA positivity, n (%)	16	(66.7)	28	(49)	0.22
*Evolution to SLE, n (%)*	4	(17)	0	(0)	0.004

Abbreviations: aCL anticardiolipin (IgM or IgG), aβ2GPI anti beta 2 glycoprotein I; SLE: systemic lupus erythematosus. P value < 0.5 calculated by Mann Whitney-test test

### IgA positivity and aPL profile

Triple aPL positivity was observed in 47.6% of IgA-positive patients and in 12.7% of IgA negative patients (*P* = 0.004), as shown in Table 3. IgA positive patients were 6 times more likely to be triple positive for aPL than IgA negative patients (adjusted OR 6.2, 95%CI 1.9–20, *P* = 0.003).

**Fig. 1:**
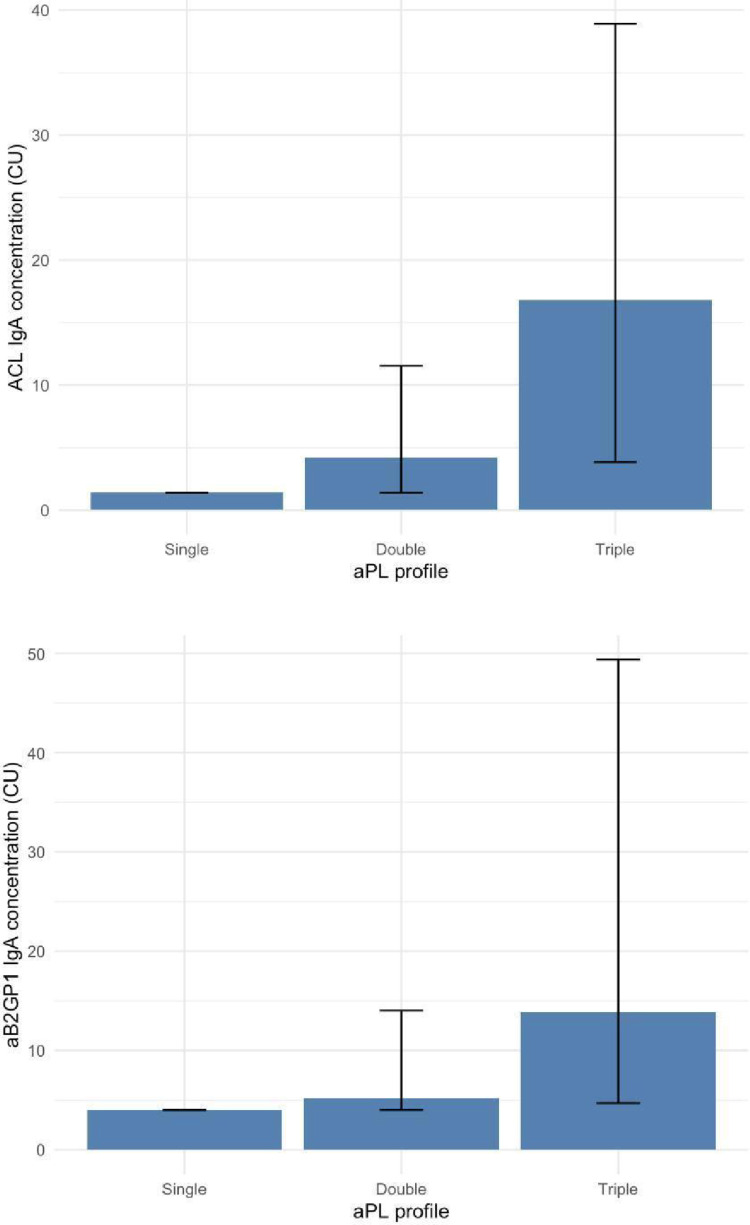
Concentration (in units of chemiluminescence) of IgA aCL and aβ2GPI by antiphospholipid profile. The graph shows the median value and the 75th percentile. The P value was calculated using the Kruskal-Wallis test. CU = units of chemiluminescence. aβ2GPI = anti-β2-glycoprotein I, aCL = anticardiolipin aPL profile was considered single positive when only LAC, IgM/IgG aCL or IgM/IgG aβ2GPI was positive, double positive when LAC plus IgM/IgG aCL/aβ2GPI were positive or when IgM/IgG aCL plus aβ2GPI were positive and triple positive when LAC, gM/IgG aCL and IgM/IgG aβ2GPI were positive

Figure 1 illustrates the distribution of IgA aCL and IgA aβ2GPI concentrations among patients with single solid-phase aPL positivity (positive IgM/IgG aCL or IgM/IgG aβ2GPI), double positivity (positive LAC and IgM/IgG aCL/aβ2GPI or positive IgM/IgG aCL and IgM/IgG aβ2GPI) or triple positive (positive LAC; IgM/IgG aCL and IgM/IgG aβ2GPI). The median IgA aCL concentration was 1.4 UC (IQ: 1.4–3.9) in single positive patients, 1.8 (IQ: 1.4–11.6) in double positive patients and 16.8 (IQ: 3.8–73.6) in triple positive patients (*P* = 0.001). The median IgA aβ2GPI concentration was 4.0 UC (IQ: 4.0–5.0) in single positive, 4.4 (IQ: 4.0–14.0) in double positive and 13.9 (IQ: 4.7-100.4) in triple positive patients (*P* = 0.001).

### IgA positivity and coagulation / inflammatory markers

Circulating levels of TNF-α were increased in IgA positive patients (median TNF-α levels: 2.11 pg/mL, IQR: 1.41–4.04) when compared to IgA negative patients (median TNF-α levels: 1.5 pg/mL, IQR: 1.14–2.53; *P* = 0.03). Circulating levels of IL-8 were also higher in IgA positive patients than in IgA negative patients. Median IL-8 levels were 29.0 pg/mL (IQ: 20.5–63.1) in IgA positive and 19.7 pg/mL (IQ: 15.4–33.0) in IgA negative patients, *P* = 0.03).

Circulating levels of TF were increased in IgA positive patients (median TF levels: 237,0 pg/mL; IQR: 190.3-402.8) when compared with IgA negative patients (median TF levels: 210.5 pg/mL; IQR: 118.8-308.1; *P* = 0.05). VWF activity was similar between IgA-positive and -negative patients, median VWF activity was 140% [IQ: 120.3-166.7]) in IgA-positive and 128% [IQ: 89.8-161.7] in IgA negative patients (*P* = 0.21).

ADAMTS-13 activity as well as interferon (INF)- alpha IL-6 circulating levels were not different between IgA-positive and IgA-negative patients (*P* = 0.95; *P* = 0.99 and *P* = 0.13; respectively). Furthermore, no statistical difference was observed between IgA-positive and IgA-negative patients regarding the concentration of C3 and C4, with respective P-values of 0.14 and 0.33. These results are shown in Table 4.

Additionally, IgA aCL and IgA aβ2GPI concentrations showed positive correlations with TF (IgA aCL: *r* = 0.26, *P* = 0.02; IgA aβ2GPI: *r* = 0.27, *P* = 0.02), IL-8 (IgA aCL: *r* = 0.30, *P* = 0.01; IgA aβ2GPI: *r* = 0.29, *P* = 0.01), and TNF-α (IgA aCL: *r* = 0.24, *P* = 0.03; IgA aβ2GPI: *r* = 0.29, *P* = 0.01). There was no correlation between coagulation or inflammatory markers and the time elapsed from the thrombotic event.

**Table 4:** Laboratory characteristics according to IgA aCL/aβ2GPI positivity

	IgA aCL/aβ2GPI positive *n* = 24		IgA aCL/aβ2GPI Negative *n* = 57		*P*
TNF-α pg/mL, median (IQR)	2.11	(1.41–4.04)	1.5	(1.14–2.53)	0.03
IL-8 pg/mL, median (IQR)	29.08	(20.52–63.12)	19.75	(15.47–33.08)	0.03
IL-6 pg/mL, median (IQR)	1.79	(1.24–3.01)	1.28	(0.74–2.98)	0.13
INF-α pg/mL, median (IQR)	16.2	(15.93–16.49)	16.24	(15.75–16.99)	0.99
TF pg/mL, median (IQR)	237.07	(190.33–402.8)	210.54	(118.82-308.18)	0.05
VWF (%), median (IQR)	140.56	(120.35-166.71)	128.02	(89.81-161.74)	0.21
ADAMTS13 (%), median (IQR)	105.12	(95.77-116.01)	107.22	(92.63-114.25)	0.95
C3 g/L, median (IQR)	1.02	(0.93–1.26)	1.19	(1.01–1.33)	0.14
C4 g/L, median (IQR)	0.18	(0.13–0.27)	0.23	(0.15–0.27)	0.33

Abbreviations: aCL anticardiolipin (IgM or IgG). aβ2GPI anti beta 2 glycoprotein I. C3 complement 3. C4 complement 4. TF: Tissue factor; VWF: Von Willebrand Factor; TF is missing in 2 patients; TNF-α pg/mL is missing in 31 patients; VWF is missing in 27 patients and ADAMTS13 is missing in 28 patients; IQR, interquartile range. P value < 0.5 calculated by Mann Whitney-test

## Discussion

In the present cohort of patients with APS and thrombosis, we observed that IgA aCL/aβ2GPI are associated with triple aPL positivity, progression to SLE and a more exacerbated inflammatory and coagulation response, highlights the association of IgA antibodies with a high-risk serological profile and potential utility of IgA testing in APS.

Detecting patients with a higher risk profile for APS is crucial for diagnosis and treatment, and remains a challenge in clinical practice. This is because APS is a disease with a heterogeneous clinical presentation, ranging from oligosymptomatic to severe thrombotic disease. Hyperlipidemia and arterial hypertension are clinical conditions associated with a higher risk of APS-related thrombosis, as suggested by the Global Antiphospholipid Syndrome Score (GAPSS) [5]. However, more specific predictive markers are needed. The most widely accepted marker of high-risk APS is aPL-TP, given its association with increased risk of thrombosis and adverse events [16]. However, detecting aPL-TP can be challenging, as aPL test results often vary significantly between laboratories due to differences in assays, reagents, equipment, and interpretation methods [14]. The absence of standardized protocols or reference plasmas to validate these tests further limits the reproducibility of aPL-TP detection across laboratories. In addition, LAC assays are highly affected by anticoagulant use [14]. While testing for LAC is not recommended in patients on anticoagulants, temporarily stopping treatment to perform the test is often not clinically feasible. Finally, high-risk APS cannot be attributed solely to the “criteria” aPL profile, highlighting the importance of identifying biomarkers associated with adverse outcomes [17]. In the present study, we demonstrated that IgA positive patients were six times more likely to present aPL-TP than those who were IgA negative, highlighting a strong association between these markers. The study focused on triple APL positivity as a marker of high-risk profile because it is more consistent and stable over time compared to LAC, which can be negatively affected by continuous anticoagulation therapy.

While IgA aCL/aβ2GPI positivity was not associated to well-known APS complications, such as recurrent thrombosis or pregnancy morbidity, we observed a higher frequency of venous thrombosis and a higher risk of progression from primary APS to SLE-associated APS among IgA-positive patients. The relationship between IgA aCL/aβ2GPI and the site of thrombosis remains controversial, with previous studies reporting conflicting findings. As an example, data by S.S. Lee et al. [18] and G. Lakos et al. [19] indicate an association between IgA aβ2GPI positivity and venous thrombosis, whereas other studies report a significant predominance of arterial thrombosis in patients with IgA aβ2GPI positivity [10, 20]. In our study, although venous thrombosis was more frequent among IgA-positive patients, the difference was not statistically significant, leaving this question open.

On the other hand, the association between IgA positivity and progression to SLE may be explained by the fact that the IgA isotype is common in patients with systemic autoimmune diseases, occurring in approximately 20% to 59% of these cases [18, 19]. Hu et al. [20] demonstrated higher positivity rates for IgA aCL and IgA aβ2GPI in patients with secondary APS compared to those with primary APS (25.42% and 20.34% versus 11.76% and 10.46%, respectively). Similarly, another recent study reported a higher prevalence of IgA aCL and IgA aβ2GPI in patients with secondary APS (59% and 47%, respectively) than in those with primary APS (42% and 41%, respectively) [10]. Progression from primary APS to SLE-associated APS occurs in approximately 10% of the patients over the course of the disease [21]. Interestingly, aPL often appears among the first detectable markers preceding the clinical onset of SLE [22]. Therefore, patients with primary APS require continuous monitoring for the development of SLE-related symptoms and signs, and testing for IgA aCL/aβ2GPI may help identify high-risk patients who should be monitored more closely.

Our results also suggest an association between IgA positivity and a thromboinflammatory response, evidenced by significant increases in TF, IL-8, and TNF-α levels. This observation is noteworthy given the growing recognition of the interplay between inflammation and coagulation in APS pathophysiology [23]. Coagulation initiation by TF is central for thrombotic complications in APS [24]. A recent study demonstrated that TF not only contributes to aPL-induced thrombosis but also promotes the persistent production of aPL. That study found that targeting TF successfully inhibited aPL development in models of latent viral infection and autoimmune disease. This inhibition prevented the aPL-induced proinflammatory and prothrombotic activation of monocytes [25]. Clinical studies demonstrated that TF is associated with aPL-TP, hypocomplementemia, increased inflammatory cytokines, and the release of neutrophil extracellular traps in patients with APS [26].

The complement and coagulation pathways are closely linked, and expanding data indicate that complement may be activated in patients with aPL and function as a cofactor in the pathogenesis of aPL-associated clinical events. Complement activation by aPL generates C5a, which induces neutrophil tissue factor-dependent procoagulant activity. Beta-2-glycoprotein I, one of the primary antigen for pathogenic aPL, has complement regulatory effects in vitro [27]. The complement cascade is considered a key player in determining the occurrence of adverse pregnancy outcomes in obstetric APS, while its role in thrombotic APS still remains unclear [13]. It is important to note that in our study there was no statistical difference between the IgA-positive and IgA-negative groups regarding the concentration of C3 and C4; however, our cohort was composed of patients with t-APS, whereas complement is expected to play a more prominent role in obstetric APS.

Additionally, a significant elevation in serum TNF-α concentration across all APS patient groups, either primary or secondary APS, was demonstrated. When compared to controls, primary APS exhibited the highest TNF-α concentrations, followed by secondary APS and SLE patients without APS [28]. A recent study demonstrated that elevated levels of TNF-α, IL-8, IL-10, sVCAM-1, sICAM-1, VEGF, IL-7, IL-12/IL-23p40, IL-4, IL-6, IL-16, PIGF, IL-23, and IL-17 A were associated not only to aPL positivity but also to overall antibody burden [29]. Nevertheless, evidence on the association between IgA aCL/aβ2GPI and markers of hypercoagulability or inflammation remains limited. In this context, our findings suggest that IgA antibodies are associated with a more inflammatory and prothrombotic APS phenotype, possibly serving as a marker of the interplay between inflammation and coagulation.

Our study has some limitations that need to be highlighted. First, the cross-sectional design prevents establishing a causal relationship; however, APS is a chronic systemic autoimmune disease where both antibody and inflammatory markers persist throughout the disease course, reflecting a stable thromboinflammatory phenotype rather than transient fluctuations. Also, while IgA was measured only once, clinical evidence suggests that IgA aCL and aβ2GPI are not transient antibodies in patients with established APS [30]. Second, although the sample size was small due to the rarity of APS, it provided sufficient statistical power to demonstrate significant differences in coagulation and inflammatory activities activity between the groups. Third, clinical data were collected from medical charts, and as a result, clinical information may have been lost during follow-up because data recording was not prospectively controlled. Fourth, the analyses were adjusted for age and sex, but not for other potential confounders such as anticoagulation and immunosuppression. Beyond the limited sample size for more comprehensive adjustment, triple aPL positivity, our primary outcome, is an inherent feature of APS and is not influenced by these treatments; therefore, their inclusion in the models is not precluded. On the other hand, anticoagulation therapy was consistent across the cohort, with the majority of patients receiving therapeutic warfarin. Immunosuppressive treatment, although prescribed only for patients with secondary APS, is expected to reduce the levels of inflammatory markers. In contrast, inflammatory markers were significantly higher in the IgA-positive group. It is possible that the levels of inflammatory markers were even higher if these patients were not receiving immunosuppression. Therefore, if the sample size permitted, further statistical adjustments would probably not change the main results. Despite this, the current study evaluated a representative cohort of patients with APS and thrombosis, with confirmed diagnoses and a well-documented clinical picture. Another strength of this study is that it is one of the few to have evaluated the association of IgA subclass antibodies with high risk APS, thereby supporting their potential role in the clinical management of patients.

In conclusion, our results demonstrate that IgA aCL and IgA aβ2GPI positivity is associated with triple aPL positivity, progression to SLE, and a thromboinflammatory profile, all of which may contribute to an increased risk of thrombosis and disease-related complications. These findings suggest the potential value of testing for IgA aCL and aβ2GPI antibodies in APS.

## Data Availability

The data that support the findings of this study are available from the corresponding author upon reasonable request.

## Abbreviations

aβ2GPI: Anti-β2 glycoprotein I
aCL: Anticardiolipin
aPL: Antiphospholipid antibodies
aPL-TP: Triple positivity antiphospholipid antibodies
APS: Antiphospholipid Syndrome
BMI: Body mass index
CIA: Chemiluminescent immunoassay
CT: Computerized tomography
CU: Units of chemiluminescence
C3: Complement 3
C4: Complement 4
dRVVT: Diluted Russell’s viper venom time
GAPSS: Global Antiphospholipid Syndrome Score
IFN-α: Interferon -alpha
IgA: Immunoglobulin A
IgG: Immunoglobulin G
IgM: Immunoglobulin M
IL: Interleukin
IQR: Interquartile range
ISTH: International Society of Thrombosis and Hemostasis
LAC: Lupus anticoagulant
MCAR: Missing Completely at Random
MR: Magnetic resonance
PIGF: Placental growth factor
SCT: Silica clotting time
SLE: Systemic lupus erythematosus
sICAM-1: Soluble Intercellular adhesion molecule-1
sVCAM-1: Soluble Vascular cell adhesion molecule-1
t-APS: Thrombotic APS
TF: Tissue Factor
TNF-α: Tumor necrosis factor-alpha
US: ultrasound
VEGF: Vascular endothelial growth factor
VWF: Von Willebrand factor
